# Organizational identity and the state of organizational identification in nursing organizations

**DOI:** 10.1002/nop2.362

**Published:** 2019-08-07

**Authors:** Naoko Tsukamoto, Akemi Hirata, Yuka Funaki

**Affiliations:** ^1^ Faculty of Human Sciences, Department of Nursing Sophia University Tokyo Japan; ^2^ College of Nursing, Department of Nursing Kanto Gakuin University Tokyo Japan

**Keywords:** hospital wards, nurses, nursing, nursing organization, organizational identification, organizational identity

## Abstract

**Aim:**

This study qualitatively identified the organizational identity of a nursing organization and determined the state of organizational identification of staff in hospital wards.

**Design:**

Cross‐sectional descriptive survey study.

**Methods:**

Interviews were conducted using interview guides; a qualitative inductive analysis was performed for the three attributes of organizational identity (central, distinctive and enduring). The study included three head nurses working in different facilities and three teams comprising three nurses each, who worked under each of the head nurses (12 nurses total).

**Results:**

Centrality comprised two subcategories: “ward work attributes” and “ward care attributes”. Clear centrality originating from a head nurse showed a strong influence on organizational culture in a hospital ward. As young staff is identified by distinctiveness in wards, it is important to clarify distinctiveness. When centrality and distinctiveness were not clear, enduring was weak.

## 1 INTRODUCTION

A survey of hospitals conduced in 2016 on the supply and demand of nurses showed stable 5‐year trends with turnover rates for full‐time nurses being 10.9% and for newly graduated nurses being 7.8% (Japan Nursing Association Public Relations Department, 2017).

This situation in nursing, where it is relatively easy for nurses to change jobs, not only leads to a shortage of staff but also has an impact on organizational culture. Okamoto (2010) validated the merits of stable employment in general corporations and noted the high morale and commitment in organizations. However, it is possible that the transience of staff lowers the morale of an organization and the commitment to an organization. In a study of nurses, Abou Hashish (2015) demonstrated that when commitment to an organization was lowered, employees showed an increased intent to leave the organization. These results suggest the possibility of a vicious circle, where the trend of transience in nursing hinders the creation of a good organizational culture, causing further desire to leave among staff. Therefore, it is necessary that each staff member has stable continuity in work duties and an attachment with the organization.

## 2 BACKGROUND

Attachment to an organization is explained in organizational research through the concept of organizational identification. Identification is a psychological term that refers to the process of establishing an identity by being in tune with others. Organizational identification theory attempts to apply this concept to organizations, proposing that organizational identification is the process by which an employee feels one with an important organization. Ashforth and Meal (1989) defined organizational identification as the recognition of a sense of belonging to or of membership in an organization. Moreover, Riketta (2005) has demonstrated that being a member of an organization is related to a cognitive, emotional self‐concept. In the case of nurses, this translates into awareness of belonging to a ward or hospital and into self‐awareness of the subjective definition of the organization, where one works, as well as of one's image, both in and outside the organization. Albert and Whetten (1985) have proposed that organizational identities are central, enduring and distinctive. The attribute of centrality is defined as being fundamental to an organization, without which it becomes a different type of organization and also loses other attributes. Distinctiveness is an attribute that differentiates an organization from others; enduring refers to the level of identity and sustainability demonstrated over time.

Kodama and Tokaji (2010)have asserted that the role of organizational identification is to increase awareness among staff regarding a desire to stay at an organization, freely cooperate with others and allow for decision‐making based on an organizational strategy in situations where choices are necessary. They have stated that organizational identifications are appropriate for explaining and predicting individual work attitudes and behaviours. Research has suggested that organizational identities deal with the identities of overall organizations and individual self‐concepts have the same attributes as those of organizational identities through identification with an organization. Pratt and Foreman (2000) indicated that identification with an organization is strengthened when the same attributes are found in both the individual and organization.

Studies on organizational identification have advanced the ways the level of identification can be empirically measured. In Japan, Takao (2013) created a measurement scale for studying major information technology corporations. However, it is difficult to quantify the details of organizational identity in each corporation; therefore, research has tended to be qualitative (Pratt & Foreman, 2000). Studies have predicted that the ward organizations of nurses have a high degree of independence (Adams & Bond, 2000) and that each ward has a different organizational identity. However, Albert and Whetten (1985) found that an organizational identity changes when the members of an organization change. Therefore, it is difficult to measure the types of organizational identities that exist and the impact that new staff has on nursing organizations that are exceptionally transient based on findings in general corporations. Thus, it is important to qualitatively examine organizational identities in hospital wards.

As Pratt and Foreman (2000) observed, a head nurse is in a position to manage diverse organizational identities, and if the head nurse uses an effective management process, it will have a major impact on an organization. Multiple studies conducted with general corporations have found that the leaders of an organization are central to organizational identity (Ravasi & Phillips, 2011). Thus, it is highly likely that the organizational identity of a head nurse in a ward's hospital organization is the source of the organizational identity of that ward. In this paper, the organizational identity of a head nurse is understood as the organizational identity of that ward. Therefore, we established the following research question for this study: “What is the organizational identity of nursing organizations at hospital wards?”

## 3 THE STUDY

This paper aims to qualitatively identify organizational identity in a ward and examine organizational identity among the nursing staff working in a ward. Moreover, the paper aims at showing the state of organizational identification in nursing organizations based on the similarities and differences found in the organizational identity of head nurses.

### 3.1 Design

Cross‐sectional descriptive survey design was used. We decided that the best way to conduct this study would be with a cross‐sectional design because we needed to collect data from head nurses and nursing staff who were working in the same environment.

### 3.2 Method

This study was conducted in Japan. Participants were selected from the staff at three hospitals in the Tokyo metropolitan area that consented to the study. Participants were head nurses and nurses of various ages who worked in the same wards as the head nurses. Nursing managers of hospitals who had given informal consent for the study were requested to recommend head nurses in wards with a good atmosphere in the three hospitals that had agreed to participate in the research.

A total of 12 participants participated: three head nurses from three separate wards and nine other nurses. The wards were an HCU/emergency ward (“HCU” below), a general surgery ward and a mixed general ward.

One‐hour interviews were held with each nurse. Individual interviews were conducted using an interview guide (Appendix 1) in spaces specified by each hospital. The interview guide contained questions regarding the organizational culture of the wards to which the head nurses and nurses belonged. Demographic data such as age and number of years worked were recorded on a form during the interview. This form was returned by mail after the interview. With the consent of the participants, interviews were recorded on a solid‐state audio recorder. The visions, nursing principles and basic policies of the hospitals were quoted from their respective websites.

### 3.3 Analysis

A verbatim of the interviews was created from the recorded data, and the portions that related to organizational identity were extracted. Those items similar to descriptions were encoded, and the head nurses and nurses performed a comparative analysis of similarities and differences in the codes using a continuous comparison method. Codes determined to be similar were compiled, placed into subcategories and systematized into the categories of central, enduring or distinctive, following Albert and Whetten (1985). Further, head nurses and nurses working in the same ward were grouped and their patterns were analysed.

To collect homogeneous data, three interviewers carefully loaded the interview guide and shared the contents before starting the interview. Interviews took place in environments where participants were able to speak freely, and before all interviews, the anonymity of the survey results and the abstention on the way were guaranteed. Three authors participated in the analysis of the text, discussing themes and subthemes, thereby increasing dependability. To ensure the credibility of the analysis, the authors continuously discussed until the end of the analysis.

### 3.4 Ethics

Prior to conducting this study, the researchers received approval from the ethics committee for “research on human subjects” at our University, with which the authors are affiliated. In obtaining cooperation from nursing managers and head nurses for their organizations, the researchers presented a research plan and ethical considerations on paper and made introductions to those individuals providing informal consent.

## 4 RESULTS

### 4.1 Attributes of respondents and characteristics of affiliated organizations

All head nurses were in their 40s, and each had worked as a nurse for about 25 years. Nursing staff members were in their 20s–40s and had worked as nurses for 6–25 years. They had worked in their current ward for 1–7 years (Tables 1 and 2).

**Table 1:** Attributes of participants included in the study

	Number	Age (years)	Years of experience	Years of employment in the current ward
Head nurse	3	40s	25	1–7
Staff	9	20–40s	6–25	2–8

**Table 2:** Characteristics of the surveyed hospitals/wards

Ward	Entity	Hospital size	Basic fee for general ward hospitalization	Organizational vision	Nursing principle	Basic policy
A (HCU)	Municipal	>500 beds	7:1	Provide safe and good quality medicine equally and fairly to protect the lives and health of citizens	Provide sincere and kind nursing for patients with varying levels of health	Actively promote efforts expected from a municipal hospital, such as “respect for patients' perspectives” and “safe management of medicine”
B (Mixed department)	Private	<300 beds	10:1	Provide safe and secure medical services Contribute to community medicine Nurture reliable medical professionals	Cultivate warm feelings, look after and support patients	(No written)
C (Surgical department)	Municipal	<200 beds	10:1	Be proud of doing one's best for patients and provide reliable medicine and welfare to ensure that people in the community can live with ease and comfort	Provide specialized care to contribute to people's life in the community by understanding the needs of patients' families	Strive to innovate and improve medicine, welfare and nursing by all staff members

### 4.2 Organizational identities of head nurses

Elements related to the “central” attribute were divided into two subcategories: “ward work attributes” and “ward care attributes”.

The “distinctive” attribute was divided into three subcategories: “goals for work initiatives”, “staff activities” and “outreach to staff”.

Under “enduring”, two subcategories were extracted: “continuity between head nurse and staff nurse” and “continuity between senior and junior staff”.

The details of the codes constituting each category are shown in Table 3.

**Table 3:** Organizational identities of head nurses

Category	Subcategory	Code
Central	Ward work	Attributes of that department (A, B and C)^a^
		Systems and work required for that department (A and B)
	Ward care	Care strived for in the ward (A, B and C)
		Current state of care (B)
Distinctive	Goals for work initiatives	A ward where staff members set their own goals (A and C)
		An attitude of working with continued awareness of the patient's perspective (A and C)
		A ward with careful time management (C)
	Staff activities	Study groups created by staff members (A and B)
		Strong desire to learn among staff members (A)
		Staff initiatives to enhance the awareness of issues (A)
		Relationships that allow for mutual follow‐up among associates (A and B)
	Outreach to staff	Taking care of every staff member (A, B and C)
		Making others aware of individual careers (A)
		Fairly allocating work (A, B and C)
		Providing information without bias (A)
		Visibly quantifying time management outcomes (C)
		Carefully maintaining work efficiency (A and C)
Enduring	Continuity between head nurses and staff	Repeatedly encourage self‐reflection (A)
		Establish a system to encourage initiatives among staff members (A)
		Convey that a leader is expected to think about the future of the ward (A)
		Discuss nursing with staff members through nursing research (A and B)
		Advice on how to get accustomed to the acquired skills (A and B)
		Feedback on positive achievements of multidisciplinary conferences (A)
	Continuity between senior and junior staffs	Leaders solicit the opinions of new staff members (A, B and C)
		An environment in which younger staff members are entrusted with giving lectures (A)
		Senior staff members nurture the perspective of junior staff members (A and B)

a Parentheses following the codes show the respondents.

### 4.3 Organizational identities of ward staff

Under the “central” attribute, 12 codes were extracted, which were then placed into the following three subcategories: “attributes of nursing”, “attributes of work” and “attributes of capabilities needed in staff”.

Under “distinctive”, two categories were extracted: “attributes of members” and “influence of the head nurse”.

Under “enduring”, three subcategories were extracted: “ward atmosphere”, “outreach to junior staff” and “systematization of guidance”.

The details of the codes constituting each category are shown in Table 4.

**Table 4:** Organizational identities of ward staff

Category	Subcategory	Code
Central	Attributes of nursing	Attributes of nursing work with regard to patient attributes
		A tense atmosphere caused by responding to emergencies
		A ward with a sense of crisis owing to the inability to determine a dramatic change
	Attributes of work	A busy ward with many patients requiring assistance with bowel movements and urination
		A ward with many older patients with cognitive impairments that makes good communication with nurses difficult
	Attributes of capabilities needed in staff members	A ward with staff members who have a desire to provide nursing in response to emergency situations
Distinctive	Attributes of members	A ward full of kind‐hearted staff
		A ward where those raising children can work
		A ward where people talk to each other
		A ward where staff members stimulate each other through individual goals
		A ward with a desire for improvement
		A ward where self‐actualization is possible
	Influence of the head nurse	Influence of the head nurse
Enduring	Ward atmosphere	A constantly stimulating environment
		An atmosphere for mutual learning
	Outreach to junior staff members	Having expectations from junior staff members
		Trusting junior staff members
		Providing guidance to junior staff members
		Allocating roles and providing opportunities to junior staff members
	Systematization of guidance	Systematization through modelling by the head nurse
		Systematization handed down from senior staff members to junior staff members

### 4.4 The relation between organizational identity of head nurses and organizational identity of staff in the same ward

Data on the head nurses and the teams of three nurses working under them in their respective wards were compiled, and these relations were examined.

#### 4.4.1 Ward A (HCU)

The head nurse listed characteristic attributes of centrality in hyper‐acute nursing, such as “a ward befitting the department (emergency care)” and “a ward where nurses have implemented triage work”. To fulfil these attributes, ward staff are actively encouraged to acquire credentials related to emergency work and lectures are held to communicate information. Centrality attributes were extracted from two primary staff members, such as “a ward with a consistent sense of urgency for responding to emergencies and a sense of preparedness for acute changes” and “a ward with a desire for nursing abilities to respond to crisis situations”. These expressions may be somewhat different, but in content, they are similar to those of the head nurse. Centrality attributes were not extracted from younger staff. Regarding distinctiveness, ward staff viewed their wards as “a place where they could express themselves” and “a place where they stimulated each other through their individual goals”. This perspective was the same for head nurses and ward staff. Regarding the attribute of enduring, head nurses aimed at transferring the skills they had acquired to staff members at the same time as actively working for continuity of cognition. The younger staff members, who received this information, saw this effort to provide them with continuity and “opened up to junior staff members about any confusion they had and proactively communicated with and brought in other staff members by asking for help”.

#### 4.4.2 Ward B (general surgery ward)

Regarding centrality, the head nurse stated the characteristics of nurses that stem from patient attributes; for example, “it is difficult to convey information on nursing because patients have a high degree of independence and are hospitalized for surgeries and tests” and “in this ward, nurses need to have the perspective of working toward a smooth surgery and then spend the rest of the day after surgery without feeling any uneasiness”. Among other means, the head nurse “creates a path by the committee” as a way of promoting this. One of the three hospital staff members said, “this ward has many independent patients as well as patients who won't be having surgery”. These were somewhat similar to the comments of the head nurse, although the other staff members at this hospital did not remark concerning centrality. Regarding “distinctiveness”, the head nurse said each nurse submits personal goals and conducts a self‐assessment and did not say anything that was different from the other wards. This mid‐level staff member was critical of the policies and methods of the head nurse. The staff member had a strong relationship with staff colleagues and had worked in that ward longer than the head nurse and, therefore, had abundant information about small efforts made by younger staff. The staff member was aware of the distinctive environment of the ward that had been handed down from the previous head nurse.

Regarding “enduring” attributes, the head nurse made no proactive efforts and followed what the previous head nurse had done. Ward staff members discussed activities for ensuring continuity of knowledge, such as “staff members pass along information in study groups and we have lively Q&A sessions” and “the atmosphere allows for voluntary participation in study groups if asked”.

#### 4.4.3 Ward C (mixed general ward)

The head nurse in this ward stated the attribute of time management regarding centrality and gave examples of efforts to quantify time management and be careful about work efficiency to manage time as ways of promoting centrality. Two of the three staff members did not say anything about centrality, although one mid‐level staff member mentioned as a nursing attribute gained from patients in the ward that “patients are elderly and have lower cognitive and awareness levels, which makes much of their communication with nurses very poor” and “in this ward, we are very busy with all the assistance we provide for bowel movements and urination”. Regarding distinctiveness, the head nurse mentioned activities for rewarding the staff's careful time management. Ward staff members mentioned the strong impact of policies created by the head nurse, such as “team members talk to each other and try to finish their work in the allotted time”, and “work is done efficiently, and everyone goes home on time”. Regarding the enduring attribute, the head nurse expressed thoughts about the ideals of establishing systems conceived and executed by nurses and having leaders make decisions and generate results. However, she gave no specific examples of actual practices. Ward staff members mentioned examples of the enduring attribute such as “when I joined the ward, everyone was nice enough to teach me when I asked, so I want to do the same for the new staff” and “my seniors told me to do things a certain way and so I will do the same”.

### 4.5 Individual differences in ward staff

In examining the comments of the nine individual ward staff members, the researchers found quantitative differences in their comments according to age. No such differences were seen for “distinctive” and “enduring”. Moreover, where the policies and thinking of the head nurses were aligned with the thinking of the ward staff, the comments of the nurses who worked in the same ward tended to be relatively similar in quality and quantity to the comments of the head nurses. However, two mid‐level staff members who did not agree with the policies of the head nurse made comments that differed qualitatively from those of the head nurse and tended to quantitatively emphasize different points.

## 5 DISCUSSION

### 5.1 Centrality in a ward organization

#### 5.1.1 Correlation between organizational visions/nursing principles and core traits of head nurses

The organizational identities of the three head nurses were evaluated in reference to the hospitals' visions, nursing principles and basic policies. The results revealed that the extracted organizational identities identified from each head nurse were influenced by the organizational visions and nursing principles of each hospital. The core traits of the organizational identity extracted from the head nurse of Ward A were consistent with the organizational visions of the hospital. The organizational visions and basic policies of Hospital A (advocating safe provision of highly sophisticated medicine and acute‐phase treatment) were congruous with the duties performed by Ward A (which is an intensive care unit). Consequently, the environment at Ward A was conducive to forming an organizational identity that was consistent with the hospital and nursing department. However, the head nurse of Ward C had visions that differed from the visions of the hospital and nursing department, with core traits peculiar to the individual being observed. While Hospital C as an organization advocates providing medical services contributing to the safe living of people in the community, the primary policy of the nursing department was the innovation in and the improvement of medicine, welfare and nursing. This suggests that the core traits of the head nurse of Ward C were shaped by the basic policy of the nursing department. The basic policies of the nursing department were originally intended to achieve the organizational vision of the hospital. However, in this case the head nurse appeared to have adopted only the basic policy, which was detached from the organizational vision. The organizational vision of the nursing department of Hospital B was more abstract and conceptual than that of the other two hospitals, with no basic policy presented for the nursing department. The fact that the head nurse of Ward B exhibited ambiguous core traits for the organizational identity could be attributed to the abstract and hard‐to‐understand nature of the nursing department's initial vision.

These results suggest that head nurses need to fully comprehend the organizational visions and nursing principles of their hospital to ensure that the identities of their ward are consistent with these visions and principles. Moreover, to ensure that the entire organization shares and functions towards the same goal, it is essential for the organization to educate the staff members on mutually understandable organizational visions, nursing principles and basic policies.

#### 5.1.2 Correlation between the characteristics of the wards and core traits of the head nurses

Takahashi and Yoshimura (2015) examined differences in stress at clinics and reported that there is greater ambiguity in roles and an insufficient sense of roles in general surgery and internal medicine wards, which leads to higher stress than ICUs and emergency rooms, surgery rooms, psychiatric wards, paediatric wards and obstetric wards. In another study, Yamauchi (1999) surveyed awareness of job characteristics in nursing and found that differences in approaches to work lead to differences in awareness of organizational culture. Moreover, clear recognition of work characteristics is strongly related to motivation and organizational behaviour. Based on these points, it is likely that the ward's clarity of nursing functions and roles differences has an impact on the clarity and quality of centrality in Ward A and the other two wards.

The two head nurses responsible for the general wards had characteristics of centrality that are of completely differing qualities, despite not having as many differences in work as the HCU. Centrality is a fundamental quality of an organization and the foundation of identity. Differences seen in the two general wards examined in this study were similar to those found in Albert and Whetten (1985). The functional attributes of wards found in Ward C, as well as the centrality related to the care attribute, are due to the strong‐willed head nurse. The present study clarified that centrality, based on the head nurse's intentions, is formed irrespective of functional and care attributes in the ward when the head nurse has uniquely strong policies and intentions for operating the ward. This study suggests that, in such cases, a change in the head nurse may result in a change in the organization even when a stable organizational culture is in place. This is an issue that affects the long‐term maturation of the ward. The high degree of independence of each ward in a hospital organization suggests that there is a need for nursing ideals to permeate into each ward in the hospital overall.

### 5.2 Organizational identification in ward organizations

Head nurses establish the direction of ward activities that embody centrality, and this direction manifests as distinctiveness in the ward. Ward staff members reflect this same distinctiveness. For example, wards with head nurses who extracted the central attribute of time management are careful in managing time and ward staff members approach this practice by viewing the following actions as distinctive: “talking to other team members and making sure work is done on time” and “working efficiently and everyone going home at the appointed time”. However, the ward with a head nurse who mentioned nursing skills required for emergency work in an HCU as an attribute of centrality had the distinctive attribute of “a ward with staff members who set challenging goals for training and obtaining credentials, such as academic conferences and study groups; take on challenges; and move forward in their efforts”. Multiple staff members had actually earned the credentials required for emergency work. It was found that the organizational identities for these types of head nurses lay the foundation for centrality and are manifested through the distinctiveness of the ward. Then, through enduring, these attributes are passed on to ward staff members. Rousseau (1998) divided organizational identification into situated identification and deep structure identification, with the former being created when those with high expectations are doing work. Individuals with this type of identification work towards a goal shared with an organization and see themselves as members of that organization. The signs of identification among nursing staff with attributes common to mixed and HCU wards match this description.

Meanwhile, the head surgical nurse whose centrality was unclear did not appear to do much to strengthen centrality or to help it continue. When centrality is vague, the direction a head nurse should take or what should continue to be done is also vague. There are three potential reasons for this. One is that a new head nurse may be in the process of forming an organizational identity and the organizational vision and nursing principle were conceptual and abstract. The final point is that in a general ward, it may be difficult to find signs of centrality. However, we may surmise that the reason such a ward has maintained a good culture is due to the foundation laid by the previous head nurse and that the new head nurse makes no changes out of respect for that. Takao (2012) showed that, among individuals who define themselves based on membership with a group, identification with an organization, or in‐group favouritism can arise even where there is no interaction and interdependence among group members, no cohesiveness and no strong leadership. In the wards in this study, we can perhaps assume there are no major fluctuations in organizational identity because even though the head nurse may not have a clear organizational identity, there is a stable foundation that has permeated among the mid‐level staff. However, even in two wards where the head nurses have broadcast a clear organizational identity, younger staff shows difficulty in conveying centrality. Accordingly, when head nurses do not broadcast an organizational identity and when mid‐level staff is transferred, there is a great possibility of disruption to the organizational identity of the ward.

### 5.3 Organizational identities of ward staff members

Concerning the organizational identities of ward staff, differences in centrality can be seen in the work experience of nurses and the organizational identities were not extracted from younger staff in the current study. Corley and Gioia (2004) surveyed 100 companies to show that there are differences in organizational identities among members, arguing that this can be due to the differences in environments inhabited by those in the higher and lower strata of organizations. They found that, at the high end, members are aware of organizational identities and relate them to specific elements with a tendency to see them as things that can be controlled. Differences in organizational identities about centrality between mid‐level and younger staff can be thought of as a similar phenomenon. However, although there are few opportunities for younger staff to directly be involved with centrality, it was found that these staff members are prepared to understand centrality by experiencing distinctiveness that leads to centrality, as discussed.

### 5.4 Diversity of organizational identities

Differences in values exist between the head nurses and certain mid‐level staff in two wards, which was reflected in differences regarding how each ward viewed centrality. In the mixed ward, a change in the head nurse caused friction between mid‐level staff and the new head nurse. In the surgery ward, mid‐level staff was hired from another hospital, which led to friction because the head nurse at their previous facility had different values than their current head nurse. Results of the present study showed that a ward may have multiple organizational identities or that friction may occur among certain staff members when a change in the head nurse or the transfer of staff occurs. Sato (2013) observed that the upper levels of an organization are strongly tied to the values of that organization and they may resist any changes to an organizational identity. This conforms to the phenomena seen in the current study. However, Kim (2011) stated, “when there is a difference between the identity presented by top management and the identity acknowledged by members of an organization, individuals in the organization will recognize the type of organization to which they belong and will follow its culture, which is a specific method for achieving visions and goals. If a culture with this pattern of thought and behavior contributes to achieving an identity, the intentional thought and behavioral patterns are institutionalized, creating and maintaining a culture”. Of the three wards analysed in the present study, the HCU had an alignment between the head nurse and mid‐level staff and their direction indicated that their activities were also aligned. There was a system of intentional continuity, with continuity between the head nurse and mid‐level staff and between mid‐level staff and younger staff. However, conditions supporting these circumstances seem to have developed over time. Staff members noted that there were strong conflicts for the first few months, after which the organizational identity acquired its current form, namely that of the head nurse and that the mid‐level staff and younger staff had a shared identity. The head nurse, as the top manager of the ward, must adjust values sufficiently among mid‐level staff when creating good organizational climate of her/his ward. The results of this study suggest that when the head nurse is successful at this, the efforts tie back into an organizational identification that includes the younger staff.

### 5.5 Overall discussion

The results of this study show that creating a stable organizational culture requires the head nurse to have a clear awareness of ward centrality and to communicate that awareness to staff. In comparing the two general wards, it was found that the influence of centrality disseminated by the head nurse was extraordinarily large, which resulted in the creation of a completely different organizational culture in the wards. Establishing a ward environment with a long‐term outlook requires a foundation that is not disrupted by the transfer of head nurses. This further necessitates that centrality be based on hospital nursing principles rather than on the individual values of the head nurse. Moreover, our results underscore how crucial it is that hospital organizations present the importance of presenting organizational visions and nursing principles that are mutually understandable to all staff members. In addition, the function of the clinic or ward has an impact on the ease with which the staff understands centrality. In general wards, with diverse patient needs, there is a higher level of ambiguity in roles compared with an HCU or ICU, surgery rooms, psychiatric wards, paediatric wards and obstetrics wards, with their special functions (Takahashi & Yoshimura, 2015), which tends to make it difficult to establish an image of centrality. However, we can assume that in wards where the head nurse has difficulty in grasping an organizational identity, particular circumstances make it more difficult to form such an identity among the nursing staff. Therefore, such wards have an even greater need for the head nurse to communicate an organizational identity. How to make a difficult‐to‐grasp organizational identity visible in a general ward is a topic for future study. Further, in creating a new ward environment, values of head nurses must sufficiently match those of mid‐level nurses, which can result in the creation of a stable organizational identity. Moreover, the study showed that younger staff identify with the ward organization through the distinctiveness of the ward before centrality is formed. Thus, it can be said that the distinctiveness of a ward is extremely important prior to understanding the essence of an organization that is its centrality. It is important for centrality to be connected to the distinctiveness of a ward and a head nurse must be aware of this when creating a ward environment.

### 5.6 Limitations

A limitation of this study is the small number of participants, with three wards that had different personnel, ward functions and nursing structures. An examination of organizational identity in these three wards is important but clarifying the overall picture will require further comparison of wards in different departments and multiple wards in the same facility, as well as further validation of the process of organizational identity formation among head nurses. Another limitation was the fact that we did not collect data on turnover rates at the surveyed hospitals and wards. Turnover is a critical piece of information when assessing organizational identity, environment and workplace relationships, a factor that needs to be explored in future studies. Further, this study was conducted in a limited region of Japan. Organizational identities and identification are likely to be affected by the underlying culture. Healee and Inada (2016) examined cultural differences in nursing organizations and indicated that Japanese nursing organizations differ from those in Western countries in the following four contexts: authority, cooperation, obligation and nursing. They argued that these cultural differences are derived from the peculiarities of Japanese culture, which emphasizes collective groups rather than individuals, prioritizes emotional subtleties over rational clarity and values seniority rather than treating all opinions equally. While these characteristics were reflected in this study and were highly likely to affect the organizational identities, they cannot be assessed based on the data available. Given that different regions may produce different organizational identities and different processes for organizational identification, we need to accumulate findings from various cultural spheres and consider the establishment of organizational environments that are suitable for each region.

## CONFLICT OF INTEREST

There are no conflicts of interest to declare.

## APPENDIX 1

### Interview guide

**Table:**

(1) What is the current atmosphere of your hospital ward? Alternatively, what kind of staff members are present?
(2) Does the atmosphere here differ from that at you previous workplaces?
(3) As the head nurse or a nurse, what do you consider as important for your ward? What changes do you want to make for your ward?
(4) Was there any particular episode that made you think that way? If yes, please describe.
(5) What specifically do you do to ensure that your ward is a comfortable/efficient place to work at? How do you try to convey such efforts to other people?
(6) When or where do you have such perceptions or thoughts regarding staff members?
(7) What is the atmosphere at ward meetings (ward conferences) and study sessions?
(8) Have you ever discussed about the atmosphere and condition in your ward with the head nurses or nurses of other departments?
(9) What do you consider to be your role (raison d'être) in your ward?
(10) What is your belief regarding the work of the head nurse (or of a nurse)?
